# Determinants of rapid weight gain in a cohort of children in the first six months of life

**DOI:** 10.1016/j.jped.2025.03.010

**Published:** 2025-05-28

**Authors:** Maíra Barros Louro Menezes, Cristina Maria Mendes Resende, Danielle Fernandes Durso, Mariane Alves Silva, Jacqueline Isaura Alvarez Leite, Sarah Aparecida Vieira Ribeiro, Juliana Farias de Novaes, Sylvia Do Carmo Castro Franceschini, Maria Tereza Cartaxo Muniz, Gustavo Velasquez-Melendez

**Affiliations:** a Universidade Federal de Minas Gerais, Departamento de Enfermagem Materno Infantil e Saúde Pública, Belo Horizonte, MG, Brazil; b Universidade Federal de Lavras, Departamento de Nutrição, Trevo Rotatório Prof. Edmir Sá Santos. Lavras, MG, CEP: 37203-202, Brasil; c Department of Neurology, Wellstone Program, University of Massachusetts Medical School, Worcester, MA, USA; d Universidade Federal de Viçosa, Departamento de Nutrição e Saúde, Viçosa, MG, Brazil; e Universidade Federal de Minas Gerais, Departamento de Bioquímica e Imunologia, Belo Horizonte, MG, Brazil; f Universidade de Pernambuco, Instituto Ciências Biológicas, Recife, PE, Brazil

**Keywords:** Birth weight, Infant, Weight gain, Polymorphism, Risk factors

## Abstract

**Objective:**

To evaluate the influence of genetic, gestational, birth, and socioeconomic factors on Rapid Weight Gain (RWG) in children between birth and six months.

**Methods:**

This is a cohort study with 267 children, information on individual and sociodemographics was obtained from the medical record. RWG was identified when the z-score difference in weight-for-age between two child assessments was > +0.67. The rs9939609 was assessed by Real-Time Polymerase Chain Reaction Taqman. The exploratory analysis of the cumulative incidence rate curves of RWG used the Kaplan-Meier, as well as the Log-Rank test to perform comparisons between the groups. To estimate the hazard ratio, the Cox semi-parametric model was used, to verify the quality of the fit of the proposed model the generalized Cox-Snell residuals were used.

**Results:**

The RWG between birth and six months was 31.84% and the incidence rate was estimated to be 2.31 cases/1000 person-days. The children who were born with inadequate weight or low weight had 1.88 times the risk of having RWG between birth and six months. In multivariate analysis, a higher risk of RWG in the first six months of life was found for children who were born weighing < 3000 g and whose mothers were overweight/obese in the pregestational phase, adjusted for the variables sex, rs 9939,609 and paternal education, rs9939609 was not associated with RWG.

**Conclusions:**

Children with lower birth weight and with mothers overweight/obese before pregnancy presented a higher risk of RWG in the first six months and rs 9939,609 was not associated with RWG.

## Introduction

The first 1,000 days of life, the period from pregnancy to a child’s second birthday – has been conceptualized as a critical window of opportunity for health and development. During this period, environmental exposure, nutritional status, and early growth trajectories have lasting effects on health outcomes throughout life [1,2]. This framework, grounded in the Developmental Origins of Health and Disease (DOHaD) hypothesis, posits that early-life experiences shape physiological and metabolic programming, influencing disease risk in later life [3]. Within this perspective, rapid weight gain (RWG) in early childhood has emerged as a key determinant of adverse health outcomes.

Several studies have consistently reported that RWG in infancy is associated with an increased risk of overweight, obesity, and altered body composition in later childhood and adulthood. Higher Body Mass Index (BMI), greater body fat percentage, total fat mass, and waist circumference have been observed in individuals who experienced RWG during infancy [3]. A recent systematic review corroborates these findings, reinforcing that RWG in the first years of life significantly elevates the likelihood of obesity persisting into adulthood [4]. These associations align with the DOHaD framework, which suggests that early excessive weight gain may induce long-term metabolic alterations, predisposing individuals to obesity and related diseases.

The risk factors for childhood RWG have been widely studied, encompassing maternal, gestational, infant, and socioeconomic [5,6,7,8]. In Brazil, for instance, evidence indicates that reduced breastfeeding duration and low socioeconomic status are among the primary contributors to RWG [9]. Moreover, a prospective cohort study identified various determinants of RWG, including paternal height, maternal nutritional status, primiparity, and maternal smoking during pregnancy [3]. These findings support the notion that RWG is not only a consequence of individual-level factors but also reflects broader contextual and intergenerational influences.

Genetic predisposition has also been implicated in RWG, though the evidence remains sparse. Notably, the rs9939609 polymorphism of the *FTO* (Fat Mass and Obesity Associated) gene has been linked to an increased risk of overweight and obesity in children and adolescents, with effects varying across populations [10,11]. Some studies suggest that this polymorphism interacts with environmental factors, such as maternal nutrition and physical activity, further modulating weight gain trajectories. Additionally, Martin et al. [12] reported an association between the FTO (rs9939609) AA genotype and gestational overweight but found no significant effect on excessive gestational weight gain or postpartum weight retention. These findings underscore the complexity of RWG etiology, which likely results from an interplay between genetic susceptibility and environmental exposures during the early critical period of growth.

Given the profound implications of RWG for long-term health, understanding its determinants is crucial for developing targeted interventions aimed at mitigating obesity risk. Therefore, this study aims to evaluate the influence of genetic, gestational, birth, and socioeconomic factors on RWG in children from a cohort followed between birth and six months of life. By adopting a theoretical framework grounded in DOHaD and integrating genetic and environmental determinants, this research seeks to contribute to the broader understanding of early-life growth patterns and their consequences for future health.

## Material and methods

### Study design and sample

This is a cohort study of children born and recruited at the maternity hospital in the municipality of Viçosa, Minas Gerais. The children evaluated in this study participated in nutritional monitoring performed in the Lactation Support Program (PROLAC) in the first six months of life.

Children selected and included in the present study had to have data collected between birth and six months of life, in addition to the genotyping result of the collected genetic polymorphisms. Therefore, initially, a total of 669 children were eligible and followed from birth to six months of life, data were collected from August 2003 to November 2007 and from December 2007 to January 2011. Between the period from August to December 2018, an active search was conducted to collect biological material for genotyping, obtaining a final sample of 267 children.

### Variables

The following child variables were used for analysis: sex, birth weight and length, gestational age at birth, type of delivery, weight and length in at least one visit until the age of six months and the rs9939609 polymorphism of the *FTO* gene genotyped. The maternal information collected included age, number of pregnancies, smoking during pregnancy, alcohol use during pregnancy, prenatal care, marital status, work outside the home, maternal education, pre-gestational BMI, gestational weight gain, and the evaluation paternal education and family income.

Weight was measured on pediatric scales with a capacity of 25 kg and sensitivity of five grams, the children were weighed without shoes and wearing light clothing. An anthropometer with an extension of one meter, divided into centimeters and subdivided into millimeters was used to measure length [13].

The weight-for-age z-score (W/A) was calculated by the WHO *Anthro* program (version 3.2.2, 2011), according to the child's date of birth, sex, and age at the time of the clinical visit. RWG was assessed and diagnosed if the difference (subtraction) of the weight-for-age z-scores between two consecutive visits was > +0.67 [3], until the child was six months old. The calculation of RWG was performed for all consecutive visits that the child had, always considering the z-score of the previous visit. Thus, the calculation of RWG could be done when the child was evaluated at 2 months and 5 months of age, for example, considering that these visits were consecutive. Since the children were evaluated continuously and RWG could happen more than once, only the first time that RWG occurred was considered. Birth weight was classified according to World Health Organization [14], considering low weight <2,500 *g*, underweight 2,500 *g* to 2,999 *g*, appropriate weight between 3,000 *g* and 3,999 *g* and macrosomia ≥4,000 *g* Regarding gestational age, 37 to 42 weeks was considered as full-term and pre-term <37 weeks [15].

Pregestational nutritional status and gestational weight gain were obtained from the pregnant woman's prenatal care card. The classification of pregestational BMI and recommended range of total weight gain during pregnancy according to pregestational nutritional status was established by the Institute of Medicine [16]. For data analysis regarding pregestational BMI, it was considered not excessive ≤ 24.99 kg/m² and excessive > 24.99kg/m².

### Collection of biological material, DNA extraction and genotyping

Children were previously instructed to rinse their oral cavity with 100 mL of water. In duplicate, the inner cheeks were scraped with a sterilized cytological brush in circular movements, with approximately 30 turns on each side. The brushes were stored in 2.0 mL screwable plastic microtubes containing absolute ethanol. The samples were stored in a refrigerator between 4 and 6 °C until extraction.

For DNA extraction the *Salting out* DNA extraction operational protocol was used [17]. After the extractions, the DNA samples were quantified in the *nanodrop* to evaluate their proper concentrations and stored in a freezer at −20 °C until the moment of genotyping.

To identify the rs9939609 polymorphism of the *FTO* gene, genotyping was performed using the *TaqMan* assay, with two differentially fluorescently labeled probes that allow detection of both alleles in a single reaction (*Applied Biosystems Inc*. (ABI), *Foster City,* CA, USA) [18]. The Polymerase Chain Reaction (PCR) *primer* and TaqMan probes was previously developed by ABI (catalog number: *FTO* locus is C_30090620_10). Assays were performed on *ABI 7900 HT Fast PCR Real Time System* (ABI), and genotype evaluations were performed using *TaqMan Genotyper Software ™* in 384-well format according to the manufacturer's instructions.

### Statistical analysis

Data from categorical variables were described by means of absolute and relative frequencies (%) and interquartile range. To verify the association between explanatory variables and RWG, the chi-square test of independence was performed. The significance level used in all tests was α <0.05.

*Hardy-Weinberg* equilibrium was assessed by the chi-square test to verify the balance of the genotype frequencies of the polymorphism studied.

Survival rates of RWG in the period from birth to six months of age were analyzed by the Kaplan-Meier method and compared by the log-rank test. The multivariate Cox proportional hazards regression model was performed to estimate hazard ratios (HRs) and 95% confidence intervals (*CIs*) of clinical variables on the risk of RWG.

To verify the quality of fit of the proposed model the generalized Cox-Snell residuals were used. In this methodology, the distribution of points on a scatter plot of estimated versus expected residuals should follow a 45-degree diagonal line.

The multivariate model followed a *Forward-driven* approach, i.e., the method of variable selections considering their potential adjustment in association with failure, the RWG. Three potential criteria for adjustment were considered: effect size >25% for the HR parameter (in modulus), i.e., increased, or decreased risk, *p*-value <0.20, and smaller confidence intervals.

As a criterion for evaluating the quality of model adjustment, the partial *likelihood* measure (*Log likelihood*) was used, thus the model with the lowest Log *likelihood* value was the model with the best multivariate adjustment. Variables with *p*-value <0.05 and a 95% confidence interval were considered significant. The analyses were conducted using Stata® software version 13.0 (StataCorp LP, College Station, TX, USA).

This study adhered to the guidelines laid down in the Declaration of Helsinki and approved by the Ethics Committee in Research with Human Beings of the Federal University of Viçosa (UFV) (protocol numbers 0119/2010; 094/2011; and 892,476/2014) CAAE: 37,866,814.3.0000.5153 and an amendment to the Ethics Committee on Human Research of the Federal University of Viçosa was submitted and approved to include polymorphisms study (protocol number 2.310.566).

## Results

The final sample was 267 children, parental, and socioeconomic variables are described in Table 1.

**Table 1:** Child, genetic, parental, and socioeconomic characteristics.

Child variables (*n*)	*n* (%)	95%CI
Sex (267)		
Male	141 (52.80)	46.77–58.76
Female	126 (47.19)	41.23–53.22
Gestational age at birth (241)		
At term (37 to 42 weeks)	229 (95.02)	91.40–97.16
Premature (<37 weeks)	12 (4.97)	02.83–08.59
Type of delivery (267)		
Normal	116 (43.44)	37.58–49.49
Cesarean section	151 (56.55)	50.50–62.41
Birth Weight (267)		
Adequate and macrosomia (≥3,000 g): - Adequate (3,000 to 3,999 g – *n* = 177) - Macrosomia (≥4,000 g – *n* = 1)	178 (66.67)	60.75–72.09
Inadequate and low weight (<3,000 g): - Inadequate (2,500 to 2,999 g – *n* = 84) - Low weight (<2,500 g – *n* = 5)	89 (33.3)	27.90–39.24
Rapid weight gain (267)		
No	183 (68.16)	62.29–73.50
Yes	85 (31.84)	26.49–37.70
SNP rs9939609 of the *FTO* gene (257)		
Absent (TT – *n* = 75)	75 (29.18)	23,91 - 35,07
Present (AT – *n* = 130 or AA – *n* = 52)	182 (70.82)	64,92 - 76,08

Mother variables (*n*)	*n* (%)	95% CI
Maternal age (264)		
Adults (≥20 years old)	223 (84.47)	79.54–88.37
Teenagers (10 to 19 years old)	41 (15.53)	11.62–20.45
Prenatal (267)		
No	1 (0.37)	0.05–2.64
Yes	266 (99.63)	97.35–99.94
Gestational smoking (262)		
No	248 (94.66)	91.15–96.82
Yes	14 (5.34)	03.17–08.84
Gestational alcohol (262)		
No	243 (92.75)	88.88–95.34
Yes	19 (7.25)	04.65–11.11
Marital status (267)		
With partner	198 (74.16)	68.53–79.08
No partner	69 (25.84)	20.91–31.46
Maternal work outside the home (267)		
Yes	122 (45.69)	39.76–51.74
No	145 (54.31)	48.25–60.23
Pregestational BMI (259)		
Not excessive (≤24.99 kg/m^2^) - Low weight (<18.5 kg/m²) (25) - Eutrophic (18.5 to 24.99 kg/m²) (188)	213 (82.24)	77.06–86.45
Excessive (>24.99 kg/m^2^)	46 (17.76)	13.54–22.93
Gestational weight gain (249)		
Not excessive	195 (78.31)	72.72–83.02
Excessive	54 (21.69)	16.97–27.27
Number of pregnancies (263)		
1 gestation	147 (55.89)	49.79–61.81
≥2 pregnancies	116 (44.11)	38.18–50.20
Maternal education (266)		
>8 years	159 (59.77)	53.72–65.53
0 to 8 years	107 (40.23)	34.46–46.27

Father variables (*n*)	*n* (%)	95% CI
Paternal Education (244)		
>8 years	120 (49.18)	42.90–55.47
0 to 8 years	124 (50.82)	44.52–57.0

Socioeconomic variables (*n*)	*n* (%)	95% CI
Family income (251)		
>1 minimum wage	168 (66,93)	60.83–72.50
≤1 minimum wage	83 (33.07)	27.49–39.16

The genotype proportions for the rs9939609 polymorphism of the *FTO* gene were in Hardy-Weinberg equilibrium (< 0,05). The *Kaplan-Meier* (*failure*) curve of the cumulative incidence of RWG in the first six months of the child's life, and the cumulative incidence of RWG was 31.84% in this complete period. The incidence estimated rate of RWG was 2.31 cases/1,000 person days (95% CI 1.87–2.86).

Univariate analysis is presented in Table 2 and only inadequate birth weight, or low birth weight (< 3,000 *g*) were associated with RWG (*p* = 0.003)*.* Children who were born with inadequate birth weight or low birth weight had an 88% higher risk of being diagnosed with RWG between birth and 6 months of life than children with adequate birth weight.

**Table 2:** Hazard ratio and CI95% of the occurrence of rapid weight gain in children evaluated from birth to 6 months of age.

Child variables	Absence of RWG % (*n*)	Presence of RWG % (*n*)	HR (95%CI)	x^2^*(p-value)*	*Log-Rank (p-value)*
Sex					
Male	67.38% (95)	32.62% (46)	Reference	0.770	0.599
Female	69.05% (87)	30.95% (39)	0.89 (0.58–1.36)		
Type of delivery					
Normal	68.97% (80)	31.03% (36)	Reference	0.806	0.973
Cesarean section	67.55% (102)	32.45% (49)	0.99 (0.64–1.52)		
Gestational age at birth^a^					
At term (≥37 to <42 weeks)	69.43% (159)	30.57% (70)			
Premature (<37 weeks)	41.67% (5)	58.33% (7)			
Birth Weight					
Adequate and macrosomia (≥3,000 g)	74.16% (132)	25.84% (46)	Reference	**0.003^a^**	**0.003^a^**
Inadequate and low weight (<3,000 g)	56.18% (50)	43.82% (39)	1.88 (1.23–2.88)		
rs9939609 of the *FTO* gene					
Absent (TT)	70.67% (53)	29.33% (22)	Reference	0.629	0.422
Present (AT or AA)	67.58% (123)	32.42% (59)	1.22 (0.74–1.99)		

Mother variables	Absence of RWG % (n)	Presence of RWG % (n)	HR (95%CI)	x^2^*(p-value)*	*Log-Rank (p-value)*
Maternal age					
Adults (≥20 years old)	67.71% (151)	32.29% (72)	Reference	0.942	0.936
Teenagers (10 to 19 years old)	68.29% (28)	31.71% (13)	1.02 (0.56–1.84)		
Number of pregnancies					
1 gestation	68.71% (101)	31.29% (46)	Reference	0.917	0.548
≥2 pregnancies	68.10% (79)	31.90% (37)	1,14 (0.74–1.76)		
Marital status					
With partner	67.17% (133)	32.83% (65)	Reference	0.555	0.455
No partner	71.01% (49)	28.99% (20)	0.82 (0.50–1.36)		
Work outside the home					
Yes	67.21% (82)	32.79% (40)	Reference	0.759	0.844
No	68.97% (100)	31.03% (45)	0.95 (0.62–1.46)		
Maternal education					
>8 years	70.44% (112)	29.56% (47)	Reference	0.388	0.428
≤8 years	65.42% (70)	34.58% (37)	1,18 (0,77–1,83)		
Pregestational BMI					
Not excessive (≤24.99 kg/m^2^)	70.42% (150)	29.58% (63)	Reference	0.121	0.098
Excessive (>24.99 kg/m^2^)	58.70% (27)	41.30% (19)	1.53 (0.91–2.56)		
Gestational weight gain					
Not excessive	66.15% (129)	33.85% (66)	Reference	0.270	0.283
Excessive	74,07% (40)	25,93% (14)	0,73 (0,41–1,30)		
Smoking in pregnancy					
No	68.95% (171)	31.05% (77)			
Yes	57.14% (8)	42.86% (6)			
Alcohol use during pregnancy					
No	68.72% (167)	31.28% (76)			
Yes	68.42% (13)	31.58% (6)			
					

Parent variables	Absence of RWG % (*n*)	Presence of RWG % (*n*)	HR (95% CI)	x^2^*(p-value)*	*Log-Rank (p-value)*
Paternal education					
>8 years	69.17% (83)	30.83% (37)	Reference	0.709	0.830
≤8 years	66.94% (83)	33.06% (41)	1.04 (0.67–1.63)		

Socioeconomic variables	Absence of RWG % (n)	Presence of RWG % (n)	HR (95%CI)	x^2^*(p-value)*	*Log-Rank (p-value)*
Family income					
>1 minimum wage	66.07% (111)	33.93% (57)	Reference	0.545	0.628
≤1 minimum wage	69.88% (58)	30.12% (25)	0.89 (0.55–1.42)		

HR (*Hazard Ratio*) obtained by univariate Cox regression; 95% CI (95% Confidence Interval) of HR; *p*-value (*Pearson*'s chi-square test); Log*-Rank* test with their respective *p*-values. The variables gestational age, smoking, alcohol, and prenatal care were not included in the analysis because of the unbalanced n between the groups that did or did not have rapid weight gain. It is considered ^a^*p*-value <0.05.

Among the independent variables selected to be part of the multivariate model, the authors excluded those that did not present a balance between the categories of presence or absence of RWG in the sample, i.e., there were zero cells or a small number of samples per cell (Table 3).

**Table 3:** *Hazard ratio* and 95%CI for the occurrence of rapid weight gain in children up to 6 months.

	Absence of RWG % (n)	Presence of RWG % (n)	Adjusted HR (95%CI)	*p-value*
Birth weight				
Appropriate weight and macrosomia (≥3,000 g)	74.16% (132)	25.84% (46)	Reference	**0.001^a^**
Inadequate weight and low weight (<3,000 g)	56.18% (50)	43.82% (39)	2.22 (1.38–3.56)	
Pregestational BMI				
Not excessive (<24.99 kg/m^2^)	70.42% (150)	29.58% (63)	Reference	**0.030^a^**
Excessive (≥24.99 kg/m^2^)	58.70% (27)	41.30% (19)	1.81 (1.05–3.12)	

Adjusted HR (*Hazard Ratio* adjusted) obtained by multivariate Cox regression; 95% CI (95% Confidence Interval); *p*-value obtained by multivariate Cox regression. Model adjusted by rs9939609 polymorphisms of the FTO (HR: 1.21 - 95% CI 0.73 - 2.03; *p*-value 0.450); paternal education (HR: 1.12 - 95% CI 0.70 - 1.78; *p*-value 0.624); and Sex (HR: 0.76 - 95% CI 0.47 - 1.22; *p*-value 0.263). It is considered ^a^*p*-value <0.05.

Although the authors acknowledge the potential relevance of including preterm children in the analysis, the number of preterm births in the sample was very small (*n* = 12). Including these cases would likely result in low statistical power to detect associations, increasing the risk of Type II error. For this reason, and to preserve the robustness of these findings, the authors chose not to include a preterm category in the final analysis.

Therefore, gestational age at birth, smoking during pregnancy, and prenatal care were excluded. Children born weighing <3,000 *g* (HR: 2.22; 95% CI, 1.38-3.56; *p* = 0.01) and whose mothers were overweight or obese before pregnancy (HR: 1.81; 95% CI 1.05 - 3.12; *p* = 0.030) had a higher risk of RWG in the first 6 months of life; these values were adjusted by sex, rs9939609 of the *FTO* gene and paternal education.

**Figure 1:**
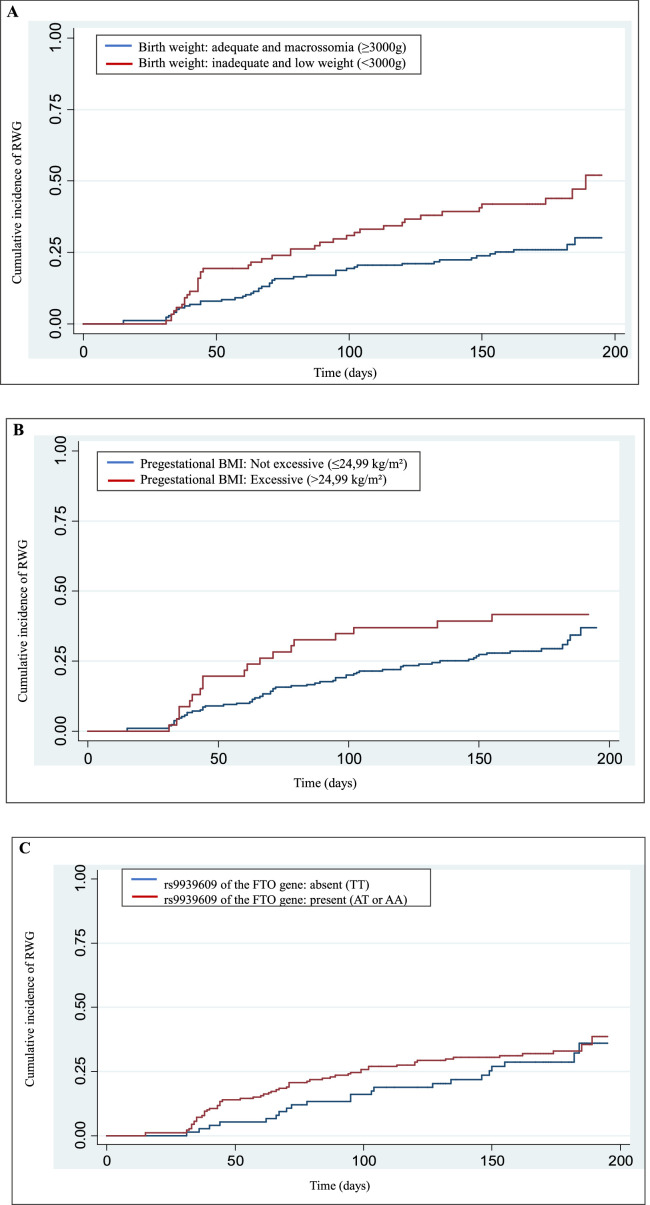
*Kaplan-Meier* curve (*failure*) *of* the cumulative incidence of rapid weight gain according to: (A) birth weight (*p*-value = 0.001), (B) pre-gestational BMI (*p*-value = 0.030), (C) rs9939609 polymorphisms of the FTO.

Figure 1 shows the *Kaplan-Meier (failure)* cumulative incidence curves of RWG according to the variables birth weight, pregestational BMI, and polymorphism. The Cox-Snell generalized residuals measure of the model, comparing the observed and predicted data, and indicates that the data are adjusted to the model.

## Discussion

In this cohort, children who were born with lower weight and whose mothers were overweight/obese pre-gestationally had a higher risk of RWG in the first six months of life. Survival analysis shows the instantaneous increased risk of RWG from birth in these indicated groups. The estimated incidence rate of RWG was 2.31 cases/1,000 person-days (95% CI, 1.87–2.86) and the cumulative incidence of RWG was 31.84% in six months of follow-up. Brazilian studies have indicated approximate occurrences, ranging from 41.9% in children until six months of age [19], 34.5% in children until 2 years of age (20), and 36.8% in children between 24 and 35 months of age [9].

The findings of this study reinforce the importance of early-life periods in shaping growth and developmental trajectories, aligning with the Developmental Origins of Health and Disease (DOHaD) framework. According to this theory, environmental, nutritional, and genetic exposures during the first 1000 days of life influence metabolic and physiological programming, with long-term health consequences [1,2]. Rapid weight gain (RWG) in infancy exemplifies this process, as it has been associated with adverse outcomes such as obesity and increased metabolic risk later in life.

A systematic review and meta-analysis demonstrated wide variations in the occurrence of RWG in various locations around the world, ranging from 12.3% to 54.2% [4].

In this study, the authors identified that children born with low birth weight (<3,000 g) had a higher risk of RWG within the first six months of life. This association may be explained by the catch-up growth mechanism, an adaptive response described in the DOHaD model, where infants with intrauterine growth restriction exhibit accelerated postnatal growth to compensate for fetal undernutrition [3,19]. While this compensatory growth may initially be beneficial, evidence suggests that excessive RWG in early infancy can lead to long-term metabolic disturbances, such as increased adiposity, insulin resistance, and higher risks of non-communicable diseases in adulthood [1,2,6]. The present study corroborates these findings, reinforcing the notion that early-life growth patterns play a critical role in health programming. In addition, other authors confirm this relationship, they explain that the occurrence of RWG appears to happen independently of birth weight [9,20,21].

Furthermore, the authors observed that infants born to mothers with pre-pregnancy overweight or obesity also had an elevated risk of RWG. This finding aligns with previous studies suggesting that maternal nutritional status before conception influences fetal metabolic programming through mechanisms such as altered placental nutrient transfer, epigenetic modifications, and changes in fetal appetite regulation [1,5]. The DOHaD hypothesis highlights that intrauterine exposure to maternal overweight/obesity can predispose offspring to rapid postnatal weight gain and increase susceptibility to obesity later in life.

A Chinese study found that children whose mothers had overweight/obese during pregnancy had a 1.15 (1.07–1.23) and 1.20 (1.11–1.30) risk of RWG between 1 and 3 months and 3 and 6 months, respectively [5]. Additionally, Subhan et al. [22] showed that infants who had RWG between birth and three months of life were twice as likely to be born to mothers who had excessive weight gain during pregnancy [22], although the authors did not find this result in the present study. Along the same lines, Nehab and colleagues [23], with the aim of evaluating whether gestational weight gain influences the body composition of full-term newborns and infants up to four months of age, observed that women with excessive weight gain had pre-higher gestational age and their newborns had greater body fat mass in kilograms and percentage than babies born to mothers with adequate or insufficient gestational weight gain, the authors found no significant difference in body composition at one, two and four months of life.

Different results on maternal influence on RWG are found in different populations, and all highlight the importance of preparation for pregnancy, as well as prenatal care. Studies reveal some factors associated with the infant RWG such as exposure to stressful life events during pregnancy [8], maternal smoking history[6] and excessive gestational weight gain [5,22]. However, infants whose mothers had insufficient gestational weight gain presented a lower risk of RWG [5].

An important aspect of this study was the inclusion of genetic analysis, specifically the rs9939609 polymorphism of the FTO gene, which has been widely associated with obesity risk in different populations. However, the authors did not find a significant association between this polymorphism and RWG. This result may be influenced by the relatively small sample size, which limits statistical power for detecting subtle genetic effects. Additionally, the interaction between genetic predisposition and environmental factors, such as maternal nutrition and early-life feeding practices, might modulate the expression of genetic risk factors related to RWG. Future studies with larger cohorts and integrative analyses incorporating gene-environment interactions are necessary to further explore these relationships. Studies showed that the rs9939609 of the FTO gene in children was associated with excessive calorie intake [24]; increased anthropometric indices, such as waist circumference [25,26], waist-to-height ratio, and waist-to-hip ratio [24]; overweight [24, 25, 26, 27, 28]; increased body fat [24,25,27,28]; and increased triglycerides [26].

The present findings highlight the importance of early-life interventions aimed at preventing excessive RWG, particularly in infants with low birth weight and those born to mothers with pre-pregnancy overweight/obesity. Strategies such as promoting exclusive breastfeeding, ensuring appropriate complementary feeding practices, and supporting maternal metabolic health before and during pregnancy may be effective in mitigating the long-term consequences of RWG. Besides lower birth weight and higher pre-pregnancy BMI were risk factors for RWG in the present study, other risk factors are also pointed out. Studies found as potential risk factors for RWG the lowest gestational ages at birth [6,9], being firstborn [6], consumption of infant formula [5,21], evening meals containing artificial milk [7], use of bottle-feeding route [7], timed infant feeding [21], and children from lower social classes [9]. A Brazilian study evaluating children between 24 and 35 months found an association between longer total breastfeeding time and lower risk of RWG;[9] however, a recent study of children in Sweden showed breastfed children for the first six months of life was negatively associated with RWG [7]. More longitudinal studies are necessary to evaluate the future impact on the health of children who presented simultaneously RWG and some genetic polymorphism associated with obesity.

A key limitation of this study is the sample size, particularly regarding genetic analyses. Studies investigating associations between genetic polymorphisms and health outcomes often require large sample sizes to detect meaningful effects. The relatively small sample in this study may have contributed to the lack of statistical significance in the genetic findings. Future research should include larger cohorts to validate these results and explore gene-environment interactions more comprehensively. Additionally, although the authors controlled potential confounders, unmeasured factors such as duration of breastfeeding and the introduction or use of infant formulas, may have influenced the observed associations.

In summary, this study provides evidence supporting the DOHaD hypothesis by demonstrating that low birth weight and maternal pre-pregnancy overweight/obesity are significant risk factors for RWG in early infancy. While no association was found between the FTO (rs9939609) polymorphism and RWG, the inclusion of genetic analysis represents an important contribution to understanding early-life obesity risk factors. Future research should focus on larger sample sizes and longitudinal designs to further elucidate the complex genetic, maternal, and early-life environmental factors influencing RWG and long-term health outcomes.

## Funding

The present study was supported by Fundação de Amparo à Pesquisa do Estado de Minas Gerais (FAPEMIG) - Finance Code APQ01777–23, Conselho Nacional de Desenvolvimento Científico e Tecnológico (CNPq) and the Coordenação de Aperfeiçoamento de Pessoal de Nível Superior (CAPES) – Finance code 001.

## Authors' contributions

**Study design:** Maíra Barros Louro Menezes, Cristina Maria Mendes Resende, Sylvia do Carmo Castro Franceschini, Gustavo Velasquez-Melendez; **Data collection:** Maíra Barros Louro Menezes, Mariane Alves Silva, Sarah Aparecida Vieira. Ribeiro; **Laboratory analysis:** Maíra Barros Louro Menezes, Cristina Maria Mendes Resende, Danielle Fernandes Durso, Mariane Alves Silva; **Data analysis:** Maíra Barros Louro Menezes, Cristina Maria Mendes Resende, Gustavo Velasquez-Melendez; **Drafting of manuscript:** Maíra Barros Louro Menezes, Cristina Maria Mendes Resende, Danielle Fernandes Durso, Mariane Alves Silva, Jacqueline Isaura Alvarez Leite, Sarah Aparecida Vieira Ribeiro, Juliana Farias de Novaes, Maria Tereza Cartaxo Muniz, Sylvia do Carmo Castro Franceschini, Gustavo Velasquez-Melendez.

## Conflicts of interest

The authors declare no conflicts of interest.
